# Our Friends from “Kaintuck”

**Published:** 1908-03

**Authors:** 


### Our Friends From "Kaintuck."

In common with all other journals, we received a communication from Dr. McCormack, Secretary of the Kentucky Medical Association, calling attention to a set of resolutions passed by said Association, and asking us to print the same, together with our position in the matter, whether we are willing to endorse these resolutions and co-operate with them in their efforts to suppress the nostrum evil. He expresses their intention to publish the replies in the *State Journal of Kentucky* in acknowledgment of this courtesy.

We must plead the always convenient editor's prerogative and decline to publish these resolutions, for lack of space, and will only say that they are practically those originally passed by the Council on Pharmacy and Chemistry of the American Medical Association.

As to our position in reference to the suppression of the nostrum evil, we cheerfully say, certainly we will help you. All we are waiting for is to know what a nostrum is, and what is not a nostrum. Just as soon as all the State societies unite on a definition of a nostrum, our editorial foot will come down on nostrums like a thousand blocks of Pennsylvania limestone.

True, the Council on Pharmacy and Chemistry has defined a nostrum, but then they don't count. They consist of a dozen or so druggists, are largely self-appointed, and in our opinion they assume (with the emphasis on the ass), entirely too much. Our hair may not have become gray in the editorial chair, but we have already worn out two cushions worth some two dollars each, and as we pay for the aforesaid we forbid them to season our broth. They say nothing in reference to the establishment of a "Home for Indigent Editors Who Assisted in the Suppression of Nostrums," and we are therefore reasonably slow in putting up our ante.

As to our refusing all advertisements except for such products as have been "Approved by the Council," etc., we must plead not guilty. We are over 21, and so are all of our subscribers; therefore, we shall continue to accept the advertisements of any legitimate pharmaceutical product, providing we feel certain that its manufacturer will pay the bill. The same applies to books and office equipment, but we always have and always will refuse to advertise whisky.

We advocate the right of any physician to prescribe what he thinks best for his patient. If any of our subscribers object to any of our advertisements, they need not renew their subscriptions at their expiration. We never fight about that. Everybody has the right to pay his money and take his choice. This is not in the Constitution, but it ought to be.

If a member of a medical association prefers a certain proprietary article, and believes that it is the best remedy for his patient, but will not prescribe it because it has not been endorsed by the Council of Druggists who decide these matters for the A. M. A., he is untrue to his calling, unjust to his patient, and unsafe as a physician.

The physician who uses an extemporaneously prepared mixture intended as a substitute for a proprietary product that requires considerable time and special apparatus to manufacture, because it is cheaper and because it meets with the approval of his society, possesses attributes and exercises self-constituted rights that a true American citizen would not tolerate in his butcher.

Of course, we don't blame you, Mac. You know where the honors fly the thickest, also the source of the ducats. You've got your job, and we've got ours. While digestion and assimilation continue active we have our own row to hoe, and with your permission we'll select the hoe. When we come to the turn in the lane, we'll all turn right if we have done our duty. All of us have at least a little light, and it's all we have to go by. If it is not brilliant enough to attract the attention of the multitude, it at least serves each one of us individually. With that with which we have been directed thus far we must go on, let it lead us where it will. As to co-operating with you gentlemen in suppressing an intangible, undefined something, in some circles known as a "nostrum," there is nothing doing.—*Albright's Office Practitioner.*

